# Fluorescent Self-Supporting Composite Film Formed from Chitosan and the Neutral Poly(3-hexylthiophene-*co*-1,4-phenylene) Polymer with Enhanced Dispersion Properties for a Small Molecule

**DOI:** 10.1021/acs.langmuir.5c00729

**Published:** 2025-04-07

**Authors:** Alessandra S. Menandro, Cornelia Bohne, Laura O. Péres

**Affiliations:** † Laboratory of Hybrid Materials, Federal University of São Paulo, Diadema, São Paulo 09913-030, Brazil; ‡ Department of Chemistry, University of Victoria, P.O. Box 1700 STN CSC, Victoria, British Columbia V8W 2Y2, Canada; § Centre for Advanced Materials and Related Technology (CAMTEC), University of Victoria, 3800 Finnerty Rd, Victoria, British Columbia V8P 5C2, Canada

## Abstract

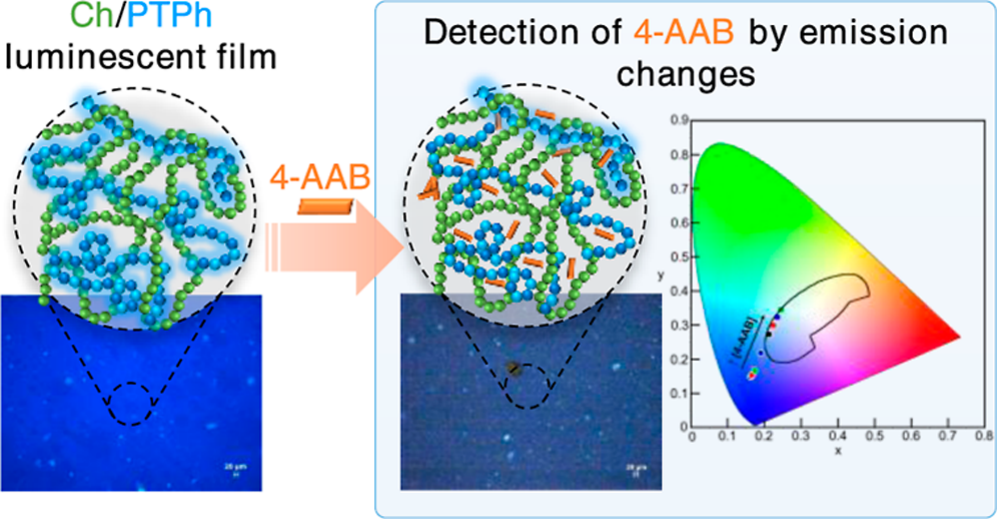

A composite film of chitosan (Ch) with a neutral conjugated polymer, poly(3-hexylthiophene-*co*-1,4-phenylene) (PTPh), was developed to combine the adsorption capacity of Ch with the fluorescence sensitivity of PTPh. Characterization of the films using thermogravimetric analysis, microscopy, and infrared, absorption, and fluorescence spectroscopies revealed that the dispersity of the target small molecule, 4-aminoazobenzene (4-AAB), was improved in the composite film compared to the pristine Ch film as evidenced in microscopy studies. In the presence of 4-AAB, the Ch/PTPh film exhibited fluorescence quenching at low 4-AAB concentrations and changes in emission spectra at higher concentrations. Photoisomerization studies suggested that the improved dispersity of 4-AAB in the composite film is due to an increase in the free volume provided by PTPh, with faster cis-to-trans isomerization observed when PTPh was present. Proof-of-concept adsorption experiments showed that the composite film adsorbed 4-AAB from an aqueous solution, leading to a change in the emission properties of the film. This qualitative characterization uncovered a dual role for the conjugated polymer in the composite film: the addition of the polymer changed the morphology and robustness of the film, and the polymer also provides the fluorophore to sense adsorbed molecules over a wide range of 4-AAB concentrations. These results show that the strategy of incorporating water-insoluble polymers at low concentrations into a versatile biopolymer leads to enhanced functionalities of a composite material.

## Introduction

Conjugated polymers, including thiophene-based polymers, have emerged as versatile materials. The polymer’s extended π-electron system delivers materials with specific electronic and optical properties for applications in organic electronics,^1^ wearable devices,^2^ colorimetric dosimeters,^3,4^ and sensors.^5−8^ The fluorescence of conjugated polymers has been instrumental for the characterization of materials and the polymer’s emission was used as a sensitive output signal for functional materials containing these polymers.^9−11^ However, challenges persist in efficiently incorporating conjugated polymers into matrices by avoiding the formation of solid-state aggregates, such as H-aggregates, that quench the emission of the polymer.^6^ Incorporating conjugated polymers into inert matrices leads to the protection and enhancement of the polymer’s luminescent properties and this strategy also provides mechanical support and film-forming ability without impacting the luminescent properties of the conjugated polymer.^12^

**Chart 1:**
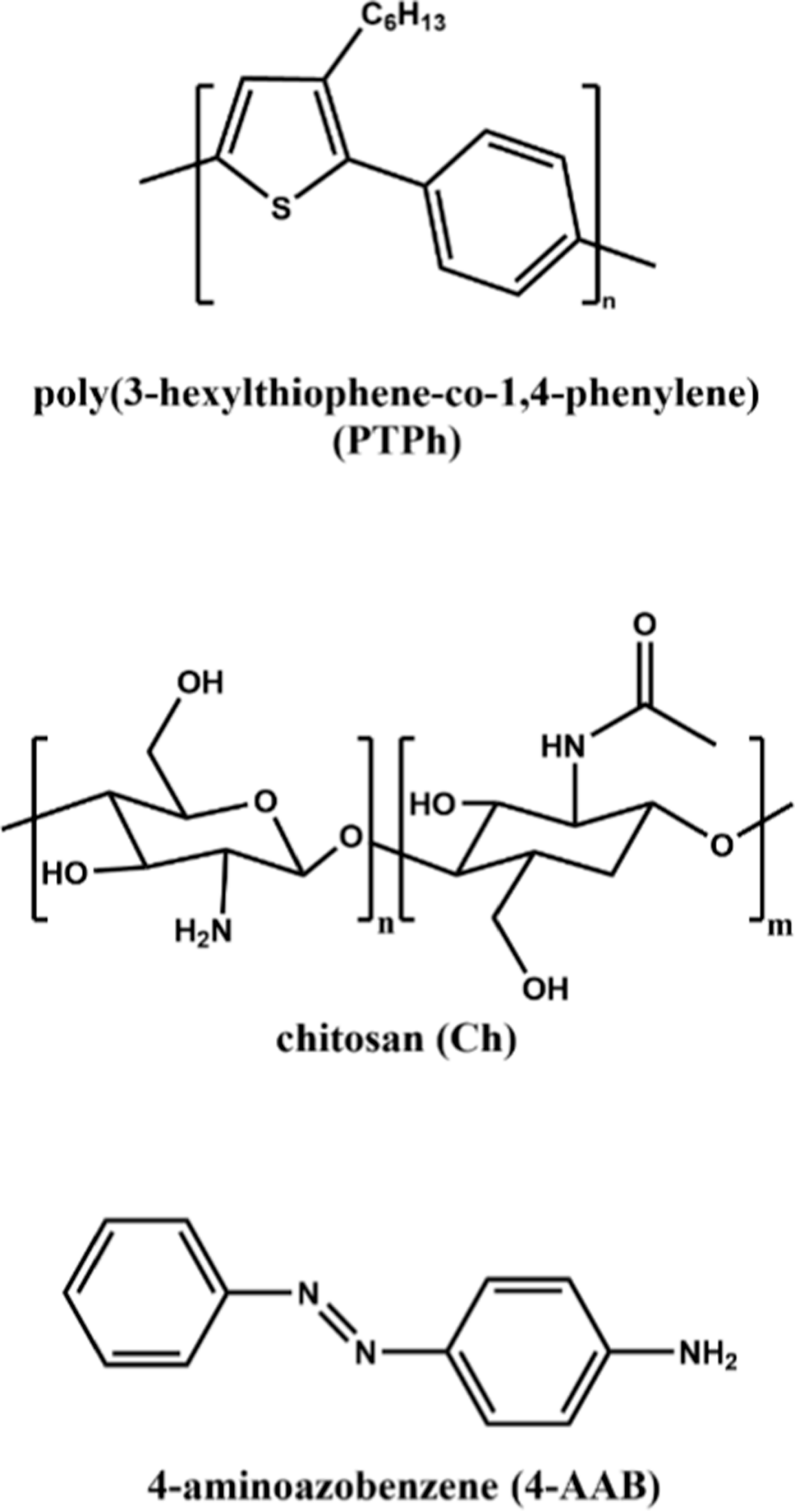
Structures of PTPh, Ch, and 4-AAB.

Among several polymers explored as matrices, chitosan (Ch, Chart 1), a natural polysaccharide derived from the deacetylation of chitin, has emerged as a sustainable material.^13,14^ The abundant availability of Ch combined with its nontoxicity, biodegradability, and easy processability decreases the production cost of functional materials containing this biopolymer.^15−17^ Ch is an excellent substrate for film formation, facilitating the incorporation of diverse materials, such as extracts,^18,19^ quantum dots,^20,21^ nanoparticles,^22,23^ or graphene.^24,25^ However, Ch films still have the drawback of low mechanical strength, high swelling, and incompatibility with some compounds, limiting the films’ performance and durability for applications^26−28^ such as in sensing and adsorption processes. Addressing these shortcomings has prompted exploration of structural modifications that add new functionalities to Ch-containing materials, including the addition of optical properties.^29−32^ Synthesis of structurally modified Ch can be complex and time-consuming and the formation of composite materials that do not require synthesis is an alternative approach to change the properties of Ch materials. Our hypothesis was that the incorporation of a neutral thiophene-based conjugated polymer, poly(3-hexylthiophene-*co*-1,4-phenylene) (PTPh, Chart 1), into Ch would render the material fluorescent and responsive to the adsorption of small molecules while also changing the swelling and adsorption properties of the material when compared to pristine Ch. This strategy was successful and showed that it can be used to overcome some of the challenges when using Ch as a polymer matrix.

PTPh is not soluble in water and this property was important to demonstrate the breadth of the methodology developed. The luminescent properties of PTPh were used as diagnostic for the presence of aggregates of PTPh and for the adsorption of a small molecule into the composite film. The dye 4-aminoazobenzene (4-AAB, Chart 1) was chosen with the objective of probing the local environment in the composite film by studying the cis-to-trans photoisomerization of 4-AAB.^33,34^ Although this objective was only partially achieved, the adsorption of 4-AAB into the Ch/PTPh films was compared to the adsorption into Ch pristine films. Our results show that the luminescent properties of PTPh, when effectively dispersed in a Ch matrix, offer insights into the superior dispersion of 4-AAB in self-supporting Ch/PTPh films. The molecular interactions among all components (Ch, PTPh, and 4-AAB) in the film lead to an enhanced dispersion of 4-AAB in the composite films. In addition, the adsorption of 4-AAB into Ch/PTPh films could be detected over a wide concentration range of 4-AAB. This is a consequence of a decrease of the emission of PTPh at low 4-AAB concentrations while at high 4-AAB concentrations, the emission spectrum of the material changes. These results show that our strategy was successful in improving the properties of a material based on a Ch matrix. This approach is instrumental for the design of functional materials where the bulk of the material is biocompatible, while the functional and value-added material, the conjugated polymer in this case, is incorporated at low loadings in the composite material. Such an approach will have impact on the development of functional materials with applications such as in sensing the adsorption of small molecules into self-supporting films.

## Experimental Section

### Materials

Glacial acetic acid (Synth, 99.7%), acetonitrile (Synth, 99.5%), ethanol (Synth, 99.9%), ultrapure Milli-Q-Plus 18.2 MΩ cm (pH ∼ 6.0) water, Ch (Sigma-Aldrich, deacetylation degree >75%, molecular mass 310,000–375,000 g mol^–1^), and 4-AAB (Sigma-Aldrich) were used as received. PTPh was synthesized by the Suzuki route (see the Supporting Information for details on the procedure, purification, and characterization).

### Ch Solution Preparation

The required amount of Ch was added to a 3 v/v % aqueous acetic acid solution to obtain a 1.0 w/v % Ch solution. The sample was left for 48 h at room temperature and without stirring, allowing the polymer to swell. Thereafter, the solution was stirred at 40 °C for 24 h. To remove small portions of particles that were not solubilized, the solution was filtered under vacuum, resulting in a clear and homogeneous solution. The portions of nonsoluble particles were very small, and therefore we can assume the solution’s concentration is close to 1 wt %.

### Preparation of Films

For the preparation of films containing PTPh, 4-AAB, or PTPh/4-AAB, the required amount of PTPh or 4-AAB was solubilized in 1 mL of acetonitrile, and then 4 mL of 1.0 w/v % Ch was added. The solutions were homogenized using a vortex mixer (ZX3 Advanced Vortex Mixer, Velp Scientifica) and transferred to a flat polypropylene Petri dish (49 × 13 mm). The films were dried at 30 °C, and after 48 h the films were peeled off. Concentrations of PTPh or 4-AAB were changed from 1.0 to 10 wt %, while for composite films containing PTPh and 4-AAB, the PTPh concentration was kept constant at 2.5 wt % and 4-AAB was changed from 0.05 to 1.0 wt %. The thickness of the films was measured with a digital micrometer. All the films had a thickness of 20 μm, except for the Ch/4-AAB film with 4-AAB concentrations between 2.5 and 10 wt %, which showed a thickness of 30 μm for 2.5 and 5.0 wt % 4-AAB, 40 μm for 7.5 wt % 4-AAB, and 100 μm for 10 wt % 4-AAB.

### Instrumentation

A Cary 60 (Agilent) UV–vis absorption spectrometer was used to measure absorption spectra (step size = 1 nm, integration = 0.1 s). All the films were placed on a solid-state support with an opening of 1.9 × 1.2 cm, and for solutions, a 10 × 10 mm quartz cell was used. The experiments were performed at room temperature.

A Fluorolog 3 (Horiba) spectrofluorimeter was used to measure steady-state fluorescence spectra (step size = 1 nm, integration = 0.1 s) using excitation and emission monochromators bandwidth of 1.5 nm for all the samples containing PTPh and 3 nm for control experiments containing 4-AAB. All the films were placed on a solid support at a 45° angle between the excitation beam and the irradiated surface. For solutions, a 10 × 10 mm quartz cell was used. Samples were excited close to the absorption maxima of PTPh (312 nm) or 4-AAB (400 nm). As a control experiment, the emission from a pristine Ch (1.0 w/v %) film was collected for excitation wavelengths of 312 and 400 nm. The experiments were performed at room temperature. The software ColorCalculator v7.77 (Osram Sylvania) was used to calculate the chromaticity coordinates and to generate a chromaticity diagram from the emission spectra of the films.

For the characterization of the films, Fourier transform infrared (FTIR) spectroscopy of all films was carried out on a IRPrestige-21 spectrometer (Shimadzu) using an attenuated total reflectance accessory with transmittance from 4000 to 400 cm^–1^ and a resolution of 4 cm^–1^. For all films, 256 scans were collected. The thermostability was established in thermogravimetric analysis (TGA, DTG-60 Shimadzu) of the films using alumina crucibles (Al_2_O_3_), where 3 ± 1 mg of the samples was heated from 25 to 800 °C at a rate of 10 °C/min under an inert atmosphere of nitrogen kept at a constant flux of 50 mL/min.

The surface of the films was analyzed using an optical microscope with a coupled fluorescence Axio Imager.A2 (Zeiss). The images were captured with a AxioCam MRc camera using an objective lens with magnification of 10×. The fluorescence images of the films containing PTPh and PTPh/4-AAB were obtained using the same objective lens and a Zeiss DAPI filter (set 49, λ_ex_ = 365 nm, λ_em_ = 445 nm). A JSM-6610LV (Jeol) scanning electronic microscope (SEM) was used to image gold coated films at a working distance of 10 mm with a 5 kV acceleration voltage and a magnification of 5000×.

### Methods

#### Photoisomerization

The photoisomerization studies were performed with Ch films containing 0.1 and 0.3 wt % of 4-AAB in the absence and presence of 2.5 wt % PTPh. A custom-built irradiation system containing a UV light emitting diode (λ = 365–370 nm, 3 W) was placed inside the UV–vis absorption spectrometer at a 5 cm distance from the film. The sample received an intensity of 0.070 mW/cm^2^ per second of light measured using a digital ultraviolet meter (H004–005, model 103, Homis). The UV light was turned on for 3 min, followed by a period during which the light was off until the initial absorbance of the film was restored or leveled off. Absorbance values were collected every 30 s to follow the photoisomerization process at the absorption maxima of 4-AAB (400 nm) and PTPh (312 nm) when the latter was present. The same photoisomerization experiment was carried out for a solution of 4-AAB (0.17 μM) in acetonitrile for comparison with the photoisomerization in the films.

#### Film Adsorption Experiments

An aqueous solution of 4-AAB (50 μM) was prepared from a 1 mM stock solution in ethanol. A piece of 1 × 1 cm (ca. 4 mg) of the Ch/PTPh (2.5 wt %) film was soaked into 10 mL of a 50 μM 4-AAB aqueous solution. The system was stirred at room temperature for 24 h with or without UV irradiation at 365 nm. The adsorption of 4-AAB into the Ch//PTPh films was monitored by UV–vis absorption spectroscopy of the 4-AAB solution after 1, 2, 4, 8, and 24 h. After 24 h, the films were removed from the solution and dried at 30 °C. Emission spectra of the Ch/PTPh film were collected before and after the 4-AAB adsorption.

## Results and Discussion

The Ch/PTPh films were characterized using several techniques to compare the properties of the composite film with those of pristine Ch films with a focus on determining the properties of the new composite material and its adsorption efficiency of the small molecule 4-AAB. The incorporation into Ch films of PTPh and 4-AAB individually or in combination did not significantly alter the thermal behavior of the Ch films and suggests that the interactions between the film’s components are physical ones. TGA was used to evaluate the thermal properties of the films (Figures S1 and S2 and Table S1). Pristine Ch films exhibited a distinctive three-step thermal degradation profile. The initial event, occurring at a temperature below 100 °C, was attributed to moisture loss, while the subsequent event corresponds to the release of adsorbed and complexed water.^35^ Lastly, the degradation and denaturalization of the polymer’s structure was observed at the highest temperature, leading to a residual mass of 30%, mainly due to the presence of mineral components.^18,36^ Pristine solid powder of PTPh also presented three distinct steps of thermal degradation. After the initial elimination of water and/or solvent, the degradation process likely starts with the smaller polymeric chains (second step) followed by the larger ones (third step), leading to a total mass loss of 100%. In contrast, the pristine solid powder of 4-AAB showed a simpler one-step degradation profile.^37^

When PTPh and 4-AAB were incorporated into Ch films, the overall thermal behavior of Ch remained largely unaltered, with all three characteristic thermal events preserved. However, there was a decrease in the *T*_onset_ of the second event for all films containing PTPh and 4-AAB individually or in combination. This reduction suggests that the adsorbed and complexed water became more superficial in the presence of PTPh and/or 4-AAB compared to pristine Ch films. The absence of new thermal events for the Ch films containing both compounds suggests that the material is a composite material formed due to intermolecular interactions between the film’s components. This assignment is supported by infrared spectroscopy data (Figure S3 and Table S2), which revealed that the bands of the composite film overlap with those of pristine Ch. No new features were observed in the infrared spectra, suggesting that no new chemical bonds were formed when PTPh, 4-AAB, or PTPh/4-AAB were incorporated into Ch films.

**Figure 1:**
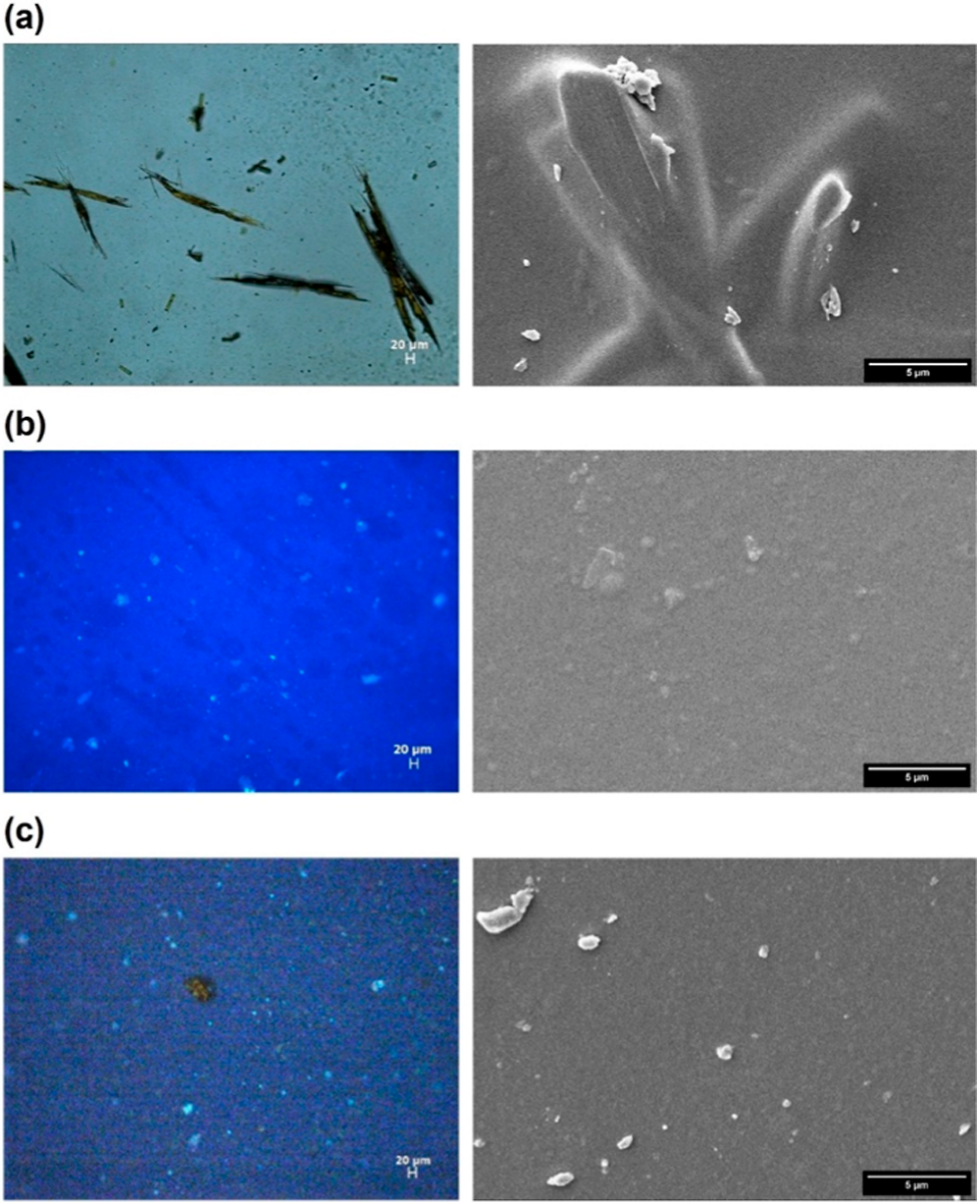
Optical microscopy (10× magnification) images (left) and SEM (5000× magnification) micrographs (right) of Ch (1.0 w/v %) films containing (a) 4-AAB (1.0 wt %), (b) PTPh (2.5 wt %), and (c) PTPh (2.5 wt %)/4-AAB (1.0 wt %). For the samples containing PTPh, the images from optical microscopy were obtained using a Zeiss DAPI filter (λ_ex_ = 365 nm), and the same microscope settings were used for all images to enable the comparison of intensities for the different films. The bars shown in the images of the left and right panels correspond to 20 and 5 μm, respectively.

Optical and SEM experiments were performed to gain information about the composite film at the microscopic level. Pristine Ch films exhibited a smooth and compact surface (Figure S4), which is characteristic of Ch matrices.^18,38^ In contrast, Ch films containing 4-AAB (Ch/4-AAB) displayed a surface with two distinct phases (Figures 1a and S5), where the phase separation between Ch and 4-AAB was observed even at low 4-AAB concentrations (Figure S6). These observations highlight the limited dispersibility of 4-AAB within Ch films, suggesting that the interactions between the 4-AAB molecules and the Ch matrix are weak.

The films containing only PTPh (Ch/PTPh, Figures 1b and S7) displayed a more homogeneous surface with the presence of some aggregates. These aggregates presented a brighter emission in the fluorescence optical microscopy experiments (Figure S7—left), suggesting that some aggregates of conjugated polymer are formed within the Ch matrix. The quantity and size of these aggregates increased with rising PTPh concentrations, with the film showing a less smooth surface with the presence 10 wt % of PTPh. This behavior indicated partial miscibility, but good compatibility of PTPh within the Ch matrix.

A homogeneous single-phase surface was obtained when 4-AAB was incorporated into Ch/PTPh films (Ch/PTPh/4-AAB films) at low 4-AAB concentrations (≤1.0 wt %). At higher concentrations of 4-AAB (Figure S8), agglomerates appeared, resulting in a rougher surface. In addition, the incorporation of 4-AAB into the film led to a decrease of the PTPh emission intensity (Figure 1b,c—left). These results show that the presence of PTPh in the Ch films increased the compatibility and dispersion of 4-AAB in the composite films when compared to the film only containing Ch, suggesting that the interactions between the components of the material are more favorable in the composite Ch/PTPh film compared to Ch films.

**Figure 2:**
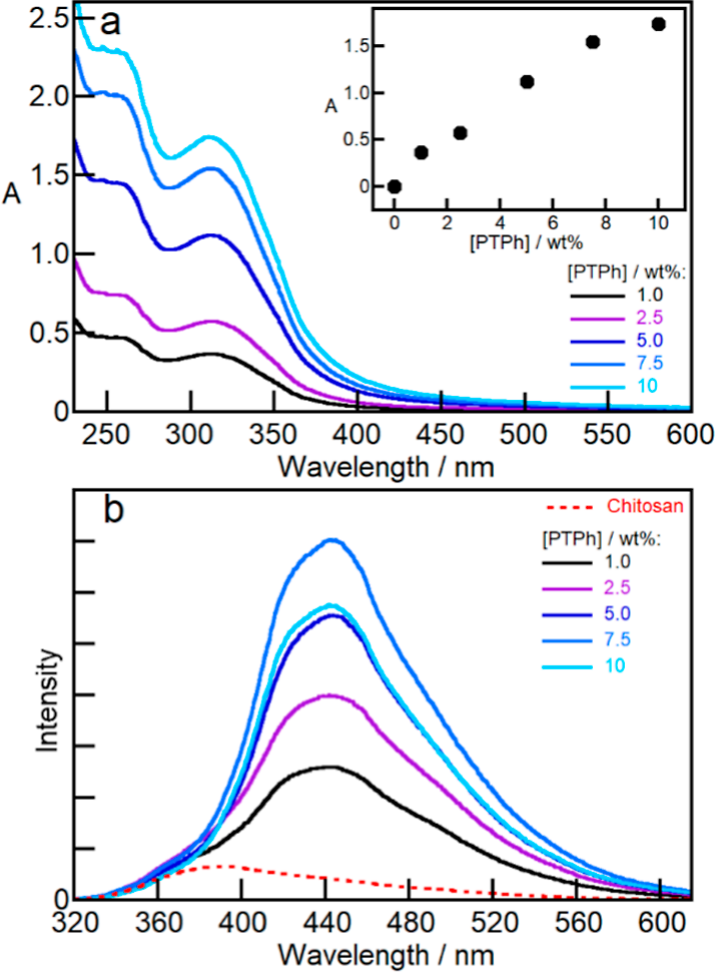
Ch (1.0 wt %) films containing PTPh (1.0–10 wt %). (a) Absorption spectra after subtraction of the Ch absorption. Inset: relationship between the absorption at 312 nm and the PTPh concentration. (b) Emission spectra (λ_ex_ = 312 nm). The red dotted line shows the emission from impurities in Ch.

Photophysical experiments were performed to provide further insights into the increased compatibility of 4-AAB in the Ch/PTPh films. These studies were based on the optical properties of the film when PTPh was present. Pristine Ch films absorb light between 220 and 400 nm (Figure S9). This absorption of Ch was subtracted from the spectra of all Ch/PTPh films to obtain the PTPh spectra when this polymer was incorporated into the film. The absorption spectra of Ch/PTPh films exhibited a maximum at 312 nm (Figure 2a) in contrast to the absorption maximum of 304 nm of PTPh in acetonitrile solutions (Figure S10). The absorbance in the films increased linearly up to a loading of 5.0 wt % of PTPh (Figure 2a). This linear trend suggests that PTPh up to a 5.0 wt % concentration was readily incorporated within the Ch matrix, leading to a good dispersion within the film.

The emission maximum at 445 nm of PTPh excited at 312 nm in the Ch/PTPh film (Figure 2b) occurs at a longer wavelength than the maximum observed at 384 nm for PTPh in acetonitrile solutions excited at 304 nm (Figure S10). The emission spectra of the Ch/PTPh films remained the same for different PTPh concentrations, except for the lower concentrations of 1.0 and 2.5 wt % where the emission from impurities in Ch broadened the emission spectra at shorter wavelengths (Figure 2b). The emission of PTPh increased at low PTPh loadings and decreased when the conjugated polymer loading was 10 wt %. The photophysics of conjugated polymers can be interpreted by considering intra- and interchain interactions.^39^ J- and H-aggregates correspond to head-to-tail and head-to-head aggregates of conjugated molecules, respectively. J-aggregates absorb at longer wavelengths than the nonaggregated molecule and J-aggregates are in general emissive. H-aggregates absorb at shorter wavelengths and their emission is suppressed.^39,40^ In the case of polymers, the interactions of chromophores within a chain lead to J-type aggregate emission, which is red-shifted compared to the monomeric emission from the polymer. In contrast, H-type aggregates are formed for interchain interactions which lead to a blue-shifted emission or a full suppression of the emission.^39^ The 80 nm red shift observed for the emission of PTPh in the Ch/PTPh film is consistent with a J-aggregate emission from the interactions of the chromophores within a single polymer chain. This result suggests that at low loadings of PTPh, the conjugated polymer chains are isolated within the Ch matrix. The decrease of the emission intensity at higher PTPh loadings is consistent with the quenching in H-aggregates formed from the interactions between chains, suggesting that the aggregation of polymer chains occurs at higher PTPh loadings.^39^

**Figure 3:**
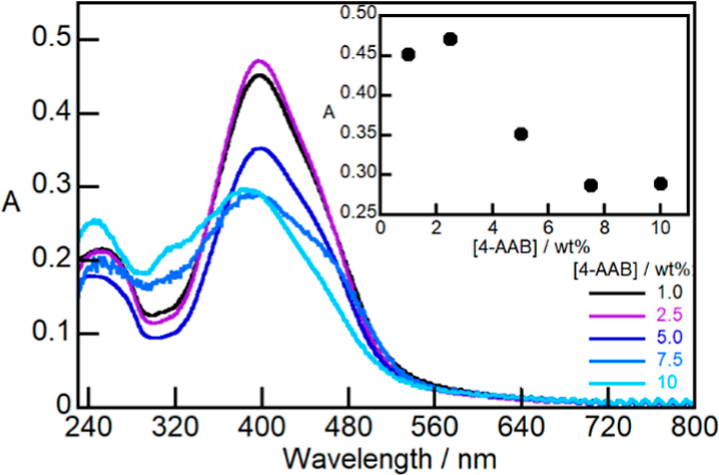
Absorption spectra of Ch films (1.0 wt %) containing 4-AAB 1.0–10 wt % after subtracting the Ch absorption. Inset: relationship between the absorption at 399 nm and the 4-AAB concentration.

**Figure 4:**
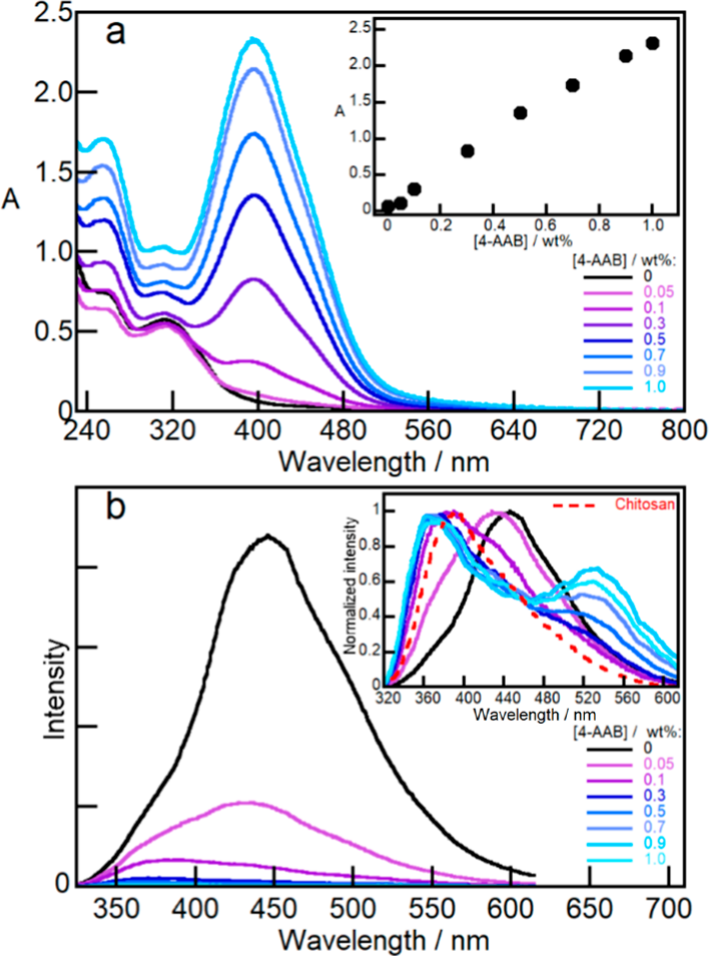
Ch (1.0 w/v %) films containing PTPh (2.5 wt %) with increasing concentrations of 4-AAB (0–1.0 wt %). (a) Absorption spectra. Inset: relationship between the absorption maximum at 396 nm and the 4-AAB concentration. (b) Emission spectra (λ_ex_ = 312 nm) Inset: normalized spectra at the maximum emission intensity.

Absorption spectra showed that 4-AAB was better dispersed in the Ch film when PTPh was present. In the absence of PTPh, 4-AAB had limited dispersibility, as evidenced by a broadening of the 4-AAB absorption spectra when its concentration in the Ch film was raised (Figure 3). This broadening combined with the decrease in the absorbance values suggests that 4-AAB aggregated within the Ch matrix. This aggregation was mitigated by the introduction of PTPh (2.5 wt %) into Ch/4-AAB films. The Ch/PTPh/4-AAB films exhibited the absorption of 4-ABB with a maximum at 396 nm, and this absorbance increased linearly up to 1.0 wt % of 4-AAB (Figure 4a).

The dispersion of 4-AAB into Ch/PTPh films led to a decrease of the PTPh emission intensity and changed the emission spectra of the films. These studies were performed at a maximum concentration of 1.0 wt % of 4-AAB and with a constant PTPh concentration of 2.5 wt %. The samples were excited at 312 nm, which corresponds to the absorption maximum of PTPh (Figure 2) and is proximate to a minimum in the 4-AAB absorption spectrum (Figure 3). Upon increasing concentrations of 4-AAB, the emission intensity at 445 nm decreased (Figure 4b), and the emission spectra broadened and split into two maxima at ca. 380 and 530 nm (inset Figure 4b).

The decrease of the PTPh emission intensity in the presence of 4-AAB is due to a combination of the quenching of the singlet excited state of PTPh by 4-AAB and the absorption of light by 4-AAB through an inner filter effect.^41^ This effect occurs because of the overlapping absorption and emission spectra of PTPh and the absorption spectra of 4-AAB. At the low concentrations of 4-AAB of 0.05 and 0.1 wt %, the absorbance of the Ch/PTPh/4-AAB films at 312 nm remained unchanged (Δ*A* ≤ 0.02) compared to the film without 4-AAB. However, the emission of PTPh decreased significantly by 4.3 and 17.7 times with the addition of 0.05 and 0.1 wt % of 4-AAB, respectively (Figure 4b). This substantial reduction of the PTPh emission intensity suggests that at these low 4-AAB concentrations, the quenching of the singlet excited state of PTPh by 4-AAB is the dominant mechanism. However, as the 4-AAB concentration exceeded 0.1 wt %, the effect on the PTPh emission became more complex, leading to a change in the emission spectra in addition to the intensity decreases (Figure 4b inset), suggesting contributions from both quenching and an inner filter effect. The appearance of the valley at 450–460 nm in the film’s emission spectra is consistent with an inner filter effect where the emitted photons are absorbed by 4-ABB. The shift of the emission maximum to shorter wavelengths is due to a combination of the inner filter effect by 4-AAB and the emission from Ch impurities. A band at ca. 530 nm was observed at higher 4-AAB concentrations, which is also present as a shoulder in the spectra at lower 4-AAB concentrations (0.05 and 0.1 wt %). This band/shoulder at 530 nm could result from the inner filter effect or be due to the presence of an additional emissive species. This additional species could be related to an excimer emission from PTPh aggregates, since excimers of thiophene/phenylene copolymers were observed to emit with a wavelength shift of 20 to 50 nm compared to the nonaggregated polymer emission.^42−45^ If present, the amount of these aggregates is minor since the emission at 530 nm was only observed when most of the PTPh emission from the dispersed PTPh chains was quenched. Excitation of Ch and Ch/PTPh films at the absorption maximum of 4-AAB of 400 nm also showed a decrease in the emission intensities as the 4-AAB concentrations were raised, which is consistent with an inner filter effect (Figures S11 and S12).

**Figure 5:**
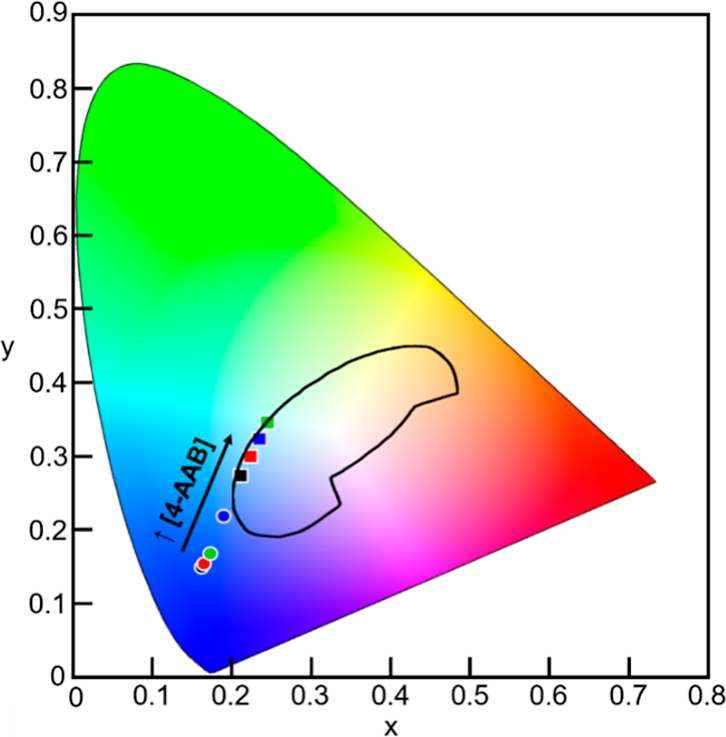
CIE 1931 chromaticity diagram for Ch (1.0 wt %)/PTPh (2.5 wt %) films (black circle) with increasing 4-ABB concentrations: 0.05 wt % (red circle), 0.1 wt % (green circle), 0.3 wt % (blue circle), 0.5 wt % (black square), 0.7 wt % (red square), 0.9 wt % (green square), and 1.0 wt % (blue square). The solid black line corresponds to the region in the chromaticity diagram that defines white light.^46,47^ The circles represent emissions of the film that are blue, while the squares correspond to white and light-green emissions. The diagram was generated using the software ColorCalculator v7.77 (Osram Sylvania).

The changes in the PTPh emission spectra are diagnostic as a change in the color of this emission when 4-AAB is added to Ch/PTPh films. In the absence of 4-AAB, the emission of PTPh films is blue as represented in a CIE 1931 chromaticity diagram (Figure S13 and Table S3), suggesting that the interactions between PTPh and Ch did not change significantly with the PTPh concentration. For example, a significant formation of PTPh aggregates would have led to a change in the emission spectra and to a change in the color of the emission. In the presence of 4-AAB in Ch/PTPh films, the color of the emission transitioned continuously from blue to white and light blue green (Figure 5). This color change serves as a second diagnostic tool for the presence of 4-AAB in the Ch/PTPh films in addition to the decrease of the emission intensity at 445 nm. The use of this color change becomes important when the PTPh emission is significantly quenched by 4-AAB and the precision in the intensity measurements at 445 nm decreases. Thus, the shift in the color of the emitted light coupled with intensity measurements serves as a diagnostic approach for discerning the presence of 4-AAB in Ch/PTPh films over a wide range of 4-AAB concentrations.

The 4-AAB photoisomerization was studied to obtain insights into the local environment for 4-AAB within Ch/PTPh/4-AAB films. In acetonitrile solutions, 4-AAB photoisomerizes from the trans- to the cis-isomer, leading to a decrease in the absorption at 382 nm (Figure S14). The cis-to-trans isomerization occurs thermally, resulting in the recovery of absorption, and this kinetics occurred over tenths of minutes for 4-AAB in acetonitrile (Figure S14). A solvent effect was reported for the rate of the cis-to-trans isomerization with the *cis* isomer of azobenzenes being stabilized in polar solvents with hydrogen bonding abilities, leading to a decrease of the activation energy for the cis-to-trans isomerization.^34,48,49^

**Figure 6:**
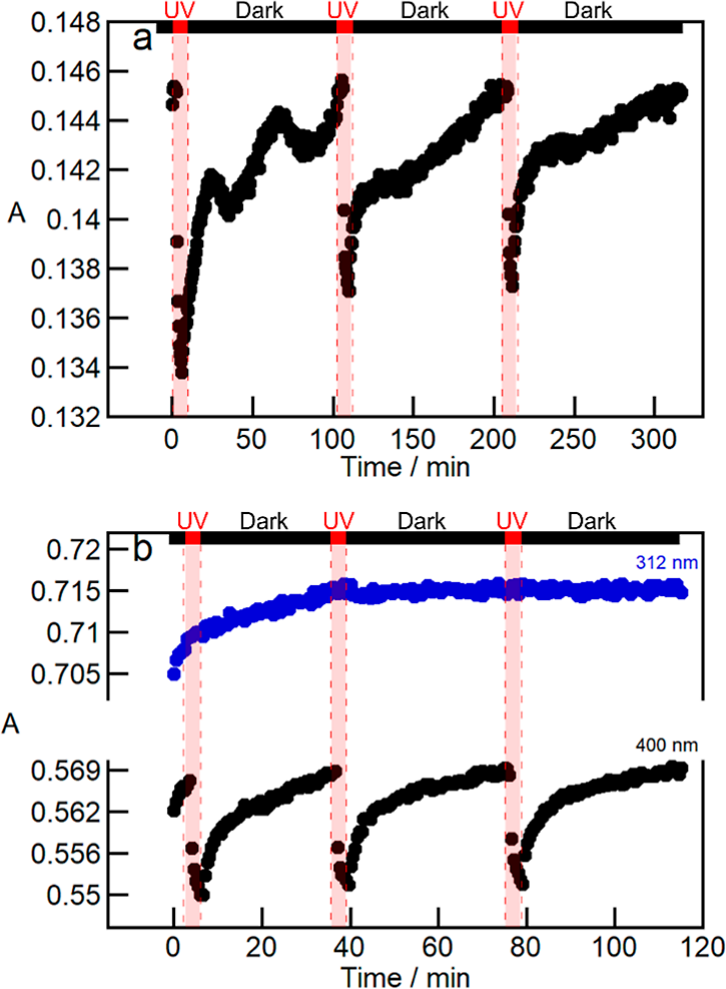
Absorbance at 400 nm (black) and 312 nm (blue) over time in the presence (red highlight) and absence of UV light (λ = 365 nm) for Ch (1.0 w/v %) films containing (a) 4-AAB (0.3 wt %) and (b) PTPh (2.5 wt %)/4-AAB (0.3 wt %).

In the films, the photoisomerization process might be limited by a lower mobility, by stronger intermolecular interactions, or by the presence of less free volume when compared to this reaction in solution.^50−52^ The photoisomerization of Ch/4-AAB and Ch/PTPh/4-AAB was investigated using a 4-AAB concentration of 0.3 wt % to ensure sufficient sensitivity for the absorption measurements. The 4-AAB isomerization was followed over time at the absorbance maxima of 4-AAB and PTPh (control experiment) for cycles in which the samples were irradiated for 3 min at 365 nm followed by a period in the dark (Figure 6). The trans-to-cis photoisomerization of 4-AAB upon irradiation was observed in the absence and presence of PTPh in the films (Figures 6 and S15 for an expanded time scale of the first cycle, and Table S4). In the case of the Ch/4-AAB films, more of the 4-AAB cis-isomer was formed during the first irradiation cycle than was formed in the two subsequent ones. For the Ch/PTPh/4-AAB films, the formation of the *cis*-isomer was similar for the three cycles (Figure 6). The 4-AAB cis-to-trans isomerization in the dark was faster when PTPh was present in the film. In addition, the kinetics was monotonous in the Ch/PTPh/4-AAB films when compared to the nonmonotonous and slower kinetics observed in the Ch/4-AAB films. This different behavior is a consequence of the formation of 4-AAB aggregates in Ch/4-AAB films while 4-AAB is well dispersed in Ch/PTPh/4-AAB films. In the Ch/4-AAB films, it is hypothesized that some form of rearrangement of the components within the film occurred during the first irradiation cycle, which likely contributed to the observed lower conversion in subsequent photoisomerization cycles. This effect probably occurs due to a decrease in mobility and free volume of 4-AAB after the rearrangement.

**Figure 7:**
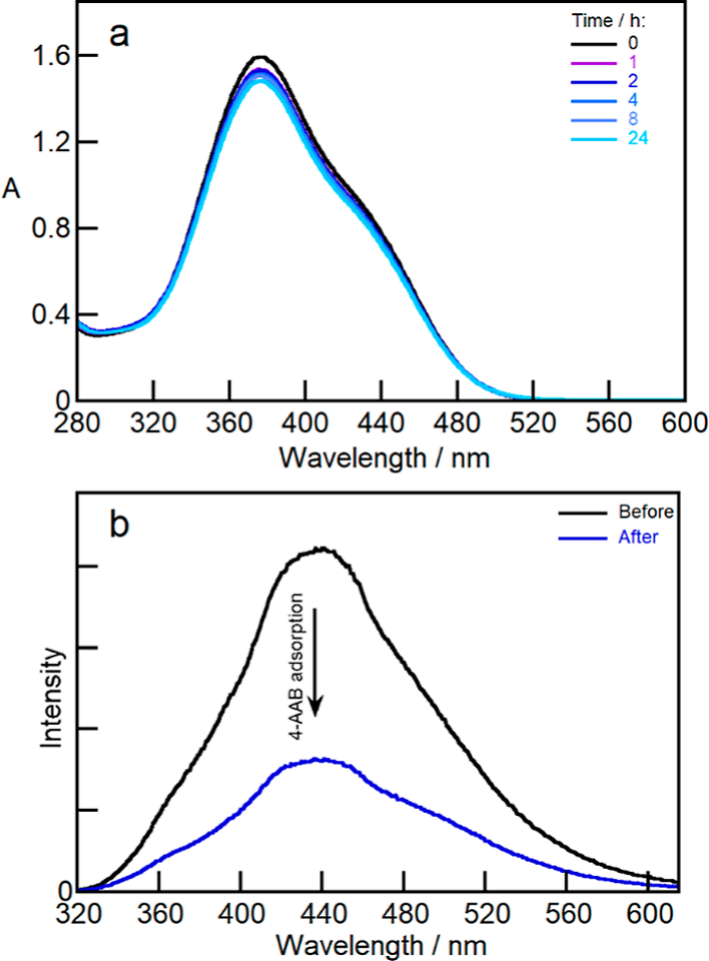
(a) Absorption spectra over time of an aqueous 4-AAB solution (50 μM initial concentration) before and after immersion of a Ch/PTPh (2.5 wt %) film. (b) Emission spectra (λ_ex_ = 312 nm) of the Ch/PTPh (2.5 wt %) film before (black) and after 24 h (blue) immersion into the 4-AAB aqueous solution.

Qualitative proof-of-concept experiments were performed to show that PTPh can be used to sense the adsorption of 4-AAB from an aqueous solution into Ch/PTPh films (Scheme S1). The Ch/PTPh films were introduced into neutral aqueous 4-AAB solutions and the changes of the absorption spectra of the aqueous solution and of the emission intensities of the Ch/PTPh films were measured (Figure 7). The decrease over time of the absorbance of the aqueous solution when the film was immersed and the quenching of the PTPh emission of the film shows that 4-AAB was adsorbed into the film. Similar decreases in absorbance were observed for Ch films in the absence of PTPh (Figure S16 and Table S5). The Ch film in the absence of PTPh swelled significantly and this film after immersion in the 4-AAB solution was fragile. In contrast, the Ch/PTPh film swelled to a lesser degree and this film was easily manipulated after drying. These qualitative experiments confirm that the PTPh emission can be used to sense the adsorption of 4-AAB into Ch/PTPh films.

The literature reports the use of conjugated polymers for sensing azo compounds in water.^53^ However, previous studies do not fabricate materials containing conjugated polymers to enable future industrial applications on sensing of azo compounds. Beyond sensing, materials capable of both sensing and adsorbing small molecules from aqueous solutions are desired. Therefore, the critical features in successfully creating a self-supporting film with high adsorption and sensing capacities for small molecules were: (i) to combine the high adsorption capacity of Ch with the fluorescence sensitivity of PTPh and (ii) creating a composite film that enhanced the dispersity of a target small molecule, 4-AAB. The amount of PTPh incorporated into the composite Ch/PTPh film was relatively small. This is important to maintain the well-defined emission from dispersed polymer chains without any significant contributions from aggregated states, thereby overcoming problems with quenching the polymer’s emission when incorporated into a matrix.^6^ While previous studies have explored Ch matrices incorporating fluorescent properties, they often rely on complex framework modifications to integrate these functionalities.^29−31^ Thus, introducing PTPh into a Ch matrix through simple intermolecular interactions without requiring synthesis is an advantageous strategy. In addition, the ability of using a small amount of the high-valued component (PTPh) has a cost benefit when these types of composite films will be explored in real-life applications. The incorporation of PTPh into Ch films improved the dispersion of 4-AAB likely because of favorable interactions between PTPh and 4-AAB^54^ and due to a change in the film’s morphology, which created a larger free volume for the incorporation of 4-AAB. This larger free volume is consistent with the shorter cis-to-trans isomerization time for 4-AAB in the Ch/PTPh films compared to Ch films where 4-AAB forms large aggregates as detected in the microscopy experiments. The dual sensing through the quenching of the PTPh emission at low 4-AAB concentrations and the changes of the emission spectra at high 4-AAB concentrations ensures that the adsorption of a broad range of 4-AAB concentrations can be determined with the same film. One possible application of composite films, such as PTPh/Ch, is in environmental sensing, where the film could be used to detect and quantify organic pollutants in water sources. The fluorescence quenching and emission changes upon adsorption of target molecules provide a convenient optical signal for real-time monitoring. This could be particularly useful for detecting aromatic amines and other hazardous compounds in industrial wastewater.

## Conclusions

A composite film of Ch with PTPh was successfully prepared, demonstrating that a water-insoluble and neutral conjugated polymer can be incorporated into a self-supporting film to combine adsorption and sensing functionalities. The incorporation of PTPh into the Ch matrix significantly enhanced the dispersion of the target small molecule 4-AAB compared to pristine Ch films. The good compatibility of PTPh with the Ch film ensured that the emission spectrum of PTPh is well-defined without the interference of the emission from aggregated states. This well-defined emission enabled the evaluation of 4-AAB dispersion into the Ch/PTPh film. Adsorption of 4-AAB led to fluorescence quenching at low 4-AAB concentrations and changes in the emission spectra and color at higher concentrations, allowing for the detection of adsorption over a wide range of 4-AAB concentrations. Proof-of-concept adsorption experiments confirmed that the composite film adsorbs 4-AAB from aqueous solutions, accompanied by fluorescence quenching in the Ch/PTPh film. The increased robustness of the Ch/PTPh films after immersion into aqueous solutions of 4-AAB compared with pristine Ch films and the ability of sensing the adsorption of molecules highlight the advantages of this strategy. Incorporating small amounts of a high-value component (PTPh) into a widely used biopolymer demonstrates a scalable and effective approach for creating multifunctional materials. The proof-of-concept adsorption experiments confirmed the film’s ability to adsorb 4-AAB from aqueous solutions while maintaining structural integrity, highlighting its potential for practical applications. The enhanced robustness of the composite film, coupled with its dual sensing mechanism, suggests that this strategy can be extended to other analytes and environmental contaminants.

## Supporting Information Available

Synthesis of PTPh; characterization and photophysics of Ch/PTPh, Ch/4-AAB, and Ch/PTPh/4-AAB films; photoisomerization of Ch/4-AAB and Ch/PTPh/4-AAB films; and adsorption experiment of 4-AAB into Ch and Ch/PTPh films (PDF)
