# Supplementary material for: Fluorescent Self-Supporting Composite Film Formed from Chitosan and the Neutral Poly(3-hexylthiophene-co-1,4-phenylene) Polymer with Enhanced Dispersion Properties for a Small Molecule
Source: Langmuir. 2025 Apr 7;41(15):10020–8. doi: 10.1021/acs.langmuir.5c00729 (PMC12020410; doi:10.1021/acs.langmuir.5c00729)
Supplement: Supplementary file 1 — la5c00729_si_001.pdf [file la5c00729_si_001.pdf]

SUPPORTING INFORMATION

Fluorescent self-supporting composite film formed from chitosan and the neutral poly(3-hexylthiophene-co-1,4-phenylene) polymer with enhanced dispersion properties for a small molecule

Alessandra S. Menandro,[§] Cornelia Bohne[†,#] and Laura O. Péres[§]*

[§] Laboratory of Hybrid Materials, Federal University of São Paulo, Diadema, SP, Brazil

[†] Department of Chemistry, University of Victoria, P.O. Box 1700 STN CSC, Victoria BC, Canada V8W 2Y2.

[#] Centre for Advanced Materials and Related Technology (CAMTEC), University of Victoria, 3800 Finnerty Rd, Victoria BC, Canada V8P 5C2.

INDEX

1. Synthesis of poly(3-hexylthiophene-co-1,4-phenylene) (PTPh) S2

2. Characterization of Ch, Ch/PTPh, Ch/4-AAB and Ch/PTPh/4-AAB films S2
Figure S1 S3
Figure S2 S4
Table S1 S5
Figure S3 S6
Table S2 S7
Figure S4 S7
Figure S5 S8
Figure S6 S9
Figure S7 S10
Figure S8 S11

3. Photophysics of Ch/PTPh, Ch/4-AAB and Ch/PTPh/4-AAB films S12
Figure S9 – S10 S12
Figure S11 S13
Figure S12 S14
Figure S13 S15
Table S3 S15

4. Photoisomerization of 4-AAB in Ch and Ch/PTPh films S16
Figure S14 S16
Figure S15 S17
Table S4 S17

5. 4-AAB adsorption into Ch/PTPh films S18
Scheme S1 S18
Figure S16 S19
Table S5 S19

References S19

1. Synthesis of poly(3-hexylthiophene-co-1,4-phenylene) (PTPh)

The polymer was synthesized using the Suzuki route[1] as previously described.[1,2] The following compounds were added to a 100 mL 3-necked flask connected to a ball-type reflux condenser: 0.588 g (3.55 mmol) of 1,4-phenylenediboronic acid (Sigma Aldrich, 95%), 654 μL (3.05 mmol) of 2,5-dibromo-3-hexylthiophene (Sigma Aldrich, 97%), 0.015 g (0.01 mmol) of the palladium catalyst $Pd[(C_6H_5)_3P]_4$ (Aldrich, 99 %), 25 mL of xylene (Synth, 98%) and 10 mL of a 2 M potassium carbonate solution (Synth, 99%). This reaction mixture was stirred and refluxed for 72 h at 90 °C under an inert nitrogen atmosphere. Thereafter, 0.124 g (0.50 mmol) of 2-bromo-3-hexylthiophene (Sigma Aldrich, 97%) was added to terminate the chain reaction and this solution was left refluxing for 24 h at 90 °C under inert nitrogen atmosphere. After this reflux, this biphasic solution was cooled to room temperature and 20 mL of hydrogen peroxide were added. The organic phase that contains the product was washed with ultra-pure Milli-Q-Plus 18.2 MΩ cm (pH~6.0) water and was separated from the aqueous phase. The organic solvent was removed by low pressure rotatory evaporation. The solid was dissolved in a minimum volume of $CHCl_3$ (Synth, 99%) and this solution was added to ca. 50 mL of methanol (Synth, 99%), leading to the precipitation of a brown solid. The yield of this synthesis was 94%. GPC (THF, polystyrene standard),: M_w= 1.6 × 10^3 g mol^{-1}, M_n/M_w= 1.53. ^1H NMR ($CDCl_3$, 300 MHz): δ 7.80-7.26 (m, 4H, Ph-H), 7.10-6.75 (m, 1H, Thiophene-H), 2.75-2.30 (m, 2H, CH_2), 1.70-1.50 (m, 2H, CH_2), 1.45-1.20 (m, 6H, CH_2), 1.00-0.80 (m, 3H, CH_3). FTIR (KBr, cm^{-1}): 3050 ($C\text{-}H_{thiop.\ ring\ stretch}$), 2927 and 2852 ($C\text{-}H_{arom.\ stretch}$), 1600 ($C = C_{arom.\ stretch}$), 1462 ($C\text{-}H_{side\ chain\ bend}$), 695 ($C\text{-}\ S\text{-}C_{thiophene\ ring\ stretch}$).

2. Characterization of Ch, Ch/PTPh, Ch/4-AAB and Ch/PTPh/4-AAB films

The thermal behaviour of all the pristine compounds and films was obtained through thermogravimetric analysis (TGA, Figure S1). The pristine Ch film and pristine PTPh powder degrade in three steps, while the 4-AAB powder degrades in one step. Despite the disparate thermal behaviour of the pristine compounds, all the films containing PTPh and/or 4-AAB exhibited a degradation profile similar to that of pristine Ch films due to the low concentration of PTPh and 4-AAB compared to Ch.

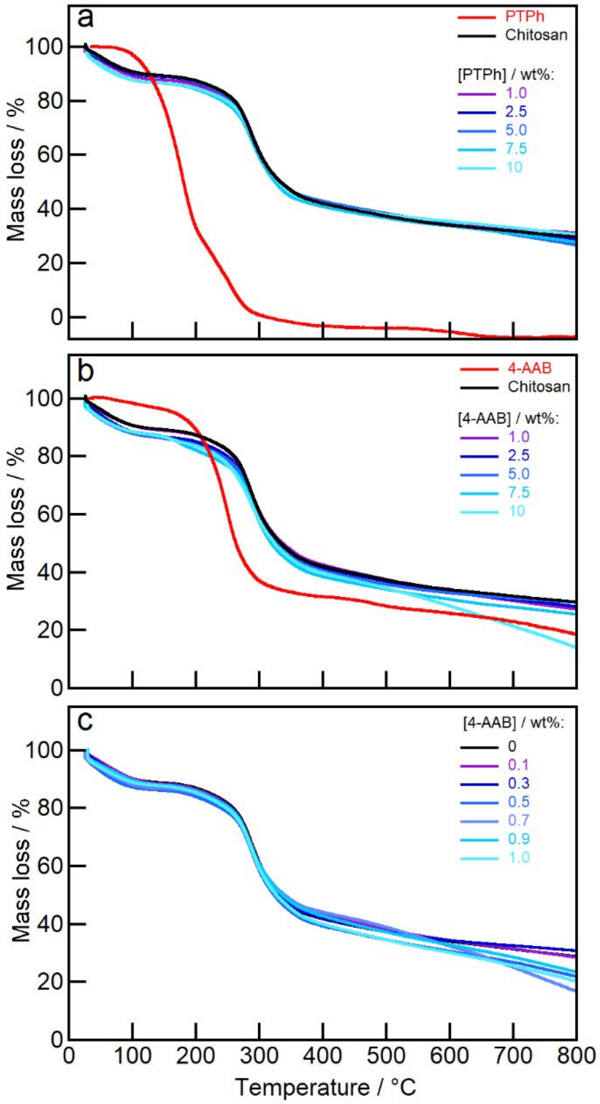

Figure S1. TGA curves (ramp from 25 to 800 °C at 10 °C/min under N_2 atmosphere with a constant flux of 50 mL/min) of (a) pristine PTPh powder, Ch films 1.0 wt% pristine and containing PTPh (1.0–10 wt%), (b) pristine 4-AAB powder, Ch films 1.0 wt% pristine and containing 4-AAB (1.0–10 wt%) and (c) Ch films 1.0 wt% containing PTPh (2.5 wt%) and 4-AAB (0–1.0 wt%).

The determination of the onset and peak temperatures is exemplified for the TGA of 4-AAB (Figure S2). The onset temperature (T_{onset}) corresponds to the temperature at which the weight loss begins, and it is determined from the intersection between the baseline and the line passing through the first inflexion point. The peak temperature (T_{peak}) is the temperature where the degradation rate is maximum, and it is determined by the peak maximum in the first derivative curve (dTG).

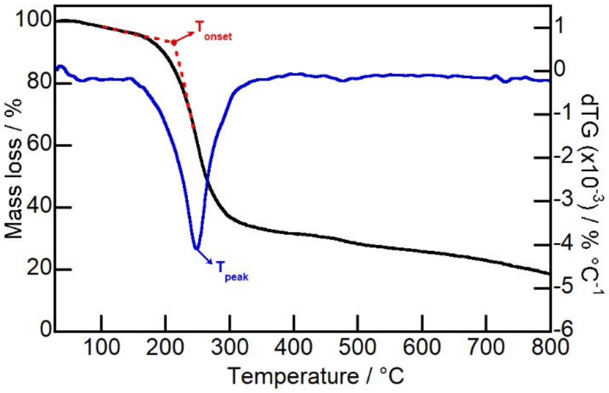

Figure S2. TGA (black) and dTG (blue) curves of pristine 4-AAB powder exemplifying the determination of T_{onset} (red) and T_{peak} (blue).

The values for T_{onset}, T_{peak} and mass loss of the pristine compounds and films are presented in Table S1. No new thermal events were observed when PTPh and/or 4-AAB were inserted into Ch film.

Table S1. T_{onset}, T_{peak} and mass loss obtained from the TGA analysis for pristine PTPh powder, pristine Azo powder, and Ch films 1 wt% pristine and containing PTPh 1–10 wt%, 4-AAB 1–10 wt%, and PTPh 2.5 wt% and 4-AAB 0–1 wt%.

Sample	1st event / °C		2nd event / °C		3rd event / °C		Mass loss / %
	T_{onset}	T_{peak}	T_{onset}	T_{peak}	T_{onset}	T_{peak}	
PTPh	135	178	195	257	570	630	100
4-AAB	213	249	-	-	-	-	81
Ch	30	56	167	190	240	285	70
Ch/PTPh / wt%							
1.0	27	56	156	195	234	285	69
2.5	30	58	155	195	235	288	71
5.0	32	60	150	195	232	286	73
7.5	27	47	148	196	225	286	72
10	28	45	150	196	228	286	69
Ch/4-AAB / wt%							
1.0	31	56	-	-	187	286	73
2.5	29	48	-	-	169	286	72
5.0	32	48	-	-	184	287	70.
7.5	28	48	124	176	228	288	75
10	31	45	134	169	190	287	86
Ch/PTPh2.5/4-AAB wt%							
0.1	28	58	162	195	238	285	72
0.3	30	56	159	195	238	285	69
0.5	30	49	153	194	235	286	78
0.7	28	56	153	194	232	286	83
0.9	32	56	150	196	232	286	77
1.0	31	56	146	196	233	287	80

Infrared spectroscopy measurements were carried out for pristine Ch, Ch/PTPh (2.5 wt%), Ch/4-AAB (2.5 wt%), and Ch/PTPh (2.5 wt%)/4-AAB (0.5 wt%) films (Figure S3). Similarly to TGA observations, films containing PTPh and 4-AAB exhibited similar spectra to the spectrum of the pristine Ch film. The PTPh and 4-AAB molecules exhibit IR vibrations in similar ranges to those of pristine Ch, resulting in the enlargement and small shifts of the bands of films containing PTPh and/or 4-AAB compared to pristine Ch films (Table S2). No new bands were observed, confirming that no new covalent bonds were formed within the films.

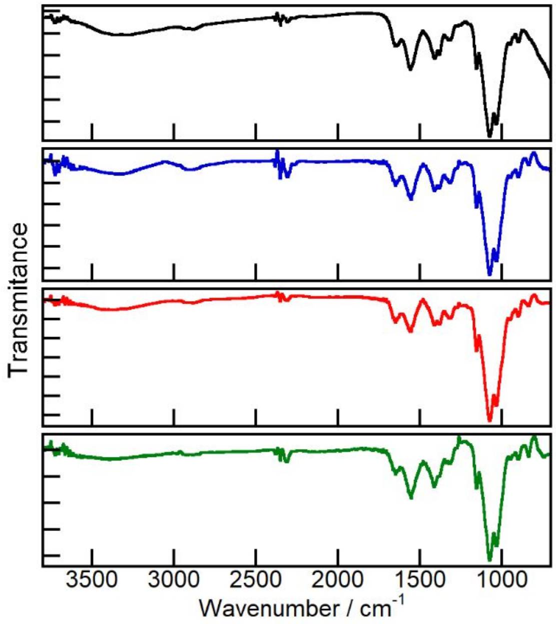

Figure S3. Infrared spectroscopy for Ch films 1.0 w/v% pristine (black) and containing PTPh (2.5 wt%) (blue), 4-AAB (2.5 wt%) (red), and PTPh (2.5 wt%)/4-AAB (0.5 wt%) (green).

Table S2. Attribution of the infrared bands (cm^{-1}) found in Ch films 1.0 w/v% pure and containing PTPh (2.5 wt%), 4-AAB (2.5 wt%), and PTPh (2.5 wt%)/4-AAB (0.5 wt%).

Attribution	Ch	Ch/PTPh	Ch/4-AAB	Ch/PTPh/4-AAB
-OH. -NH_2 stretch	3341	3340	3365	3366
C-H asym stretch	2930	2960	2945	2928
C-H sym stretch	2883	2875	2880	2866
C=O amide I stretch	1649	1645	1637	1645
N-H amide II stretch	1555	1556	1576	1553
CH_2 bending	1408	1408	1410	1410
CH. CH_3 bending	1385	1381	1381	1377
C-N amide III bending	1317	1317	1328	1315
O-H bending	1256	1260	1263	1246
C-O-C sym stretch	1153	1153	1153	1153
C-O (C_3-OH) bending	1074	1074	1070	1072
C-O (C_6-OH) bending	1029	1030	1030	1032

Optical and scanning electron microscopy (SEM) measurements were performed to evaluate the dispersion of PTPh and 4-AAB within Ch films. The pristine Ch film exhibits a smooth and compact surface of only one phase (Figure S4). Since Ch does not exhibit intense fluorescence, images with a DAPI filter (λ_{ex}= 365 nm) were not possible to be collected.

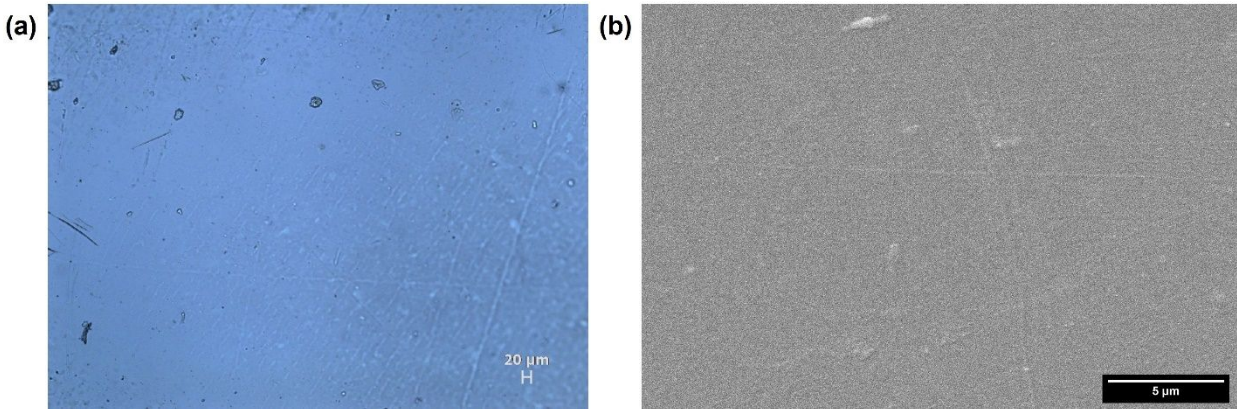

Figure S4. (a) Optical microscopy images (objective lens with magnification of 10×) and (b) SEM (5000× magnification) micrographs of pristine Ch films 1.0 w/v%. No features are seen in (a) and (b), and the image in (a) has no fluorescence.

Two phases were observed for Ch/4-AAB films (Figure S5). Distinct features were observed for all films, increasing in quantity with the increase in the concentration of 4-AAB. Phase separation was observed for 4-AAB concentrations as low as 0.3 wt% (Figure S6), suggesting the low dispersibility of 4-AAB within Ch films which suggests that the 4-AAB–Ch interactions are weak.

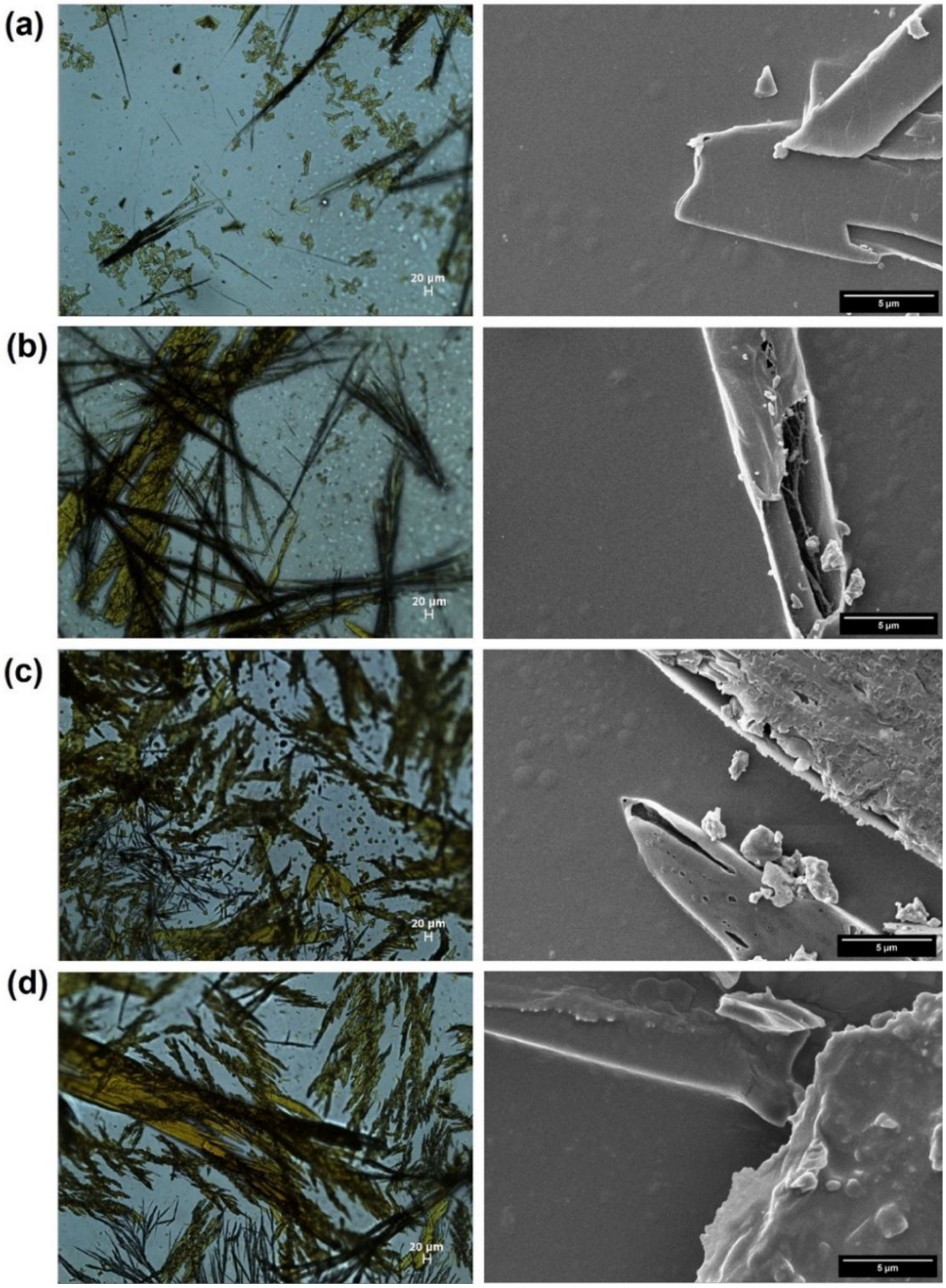

Figure S5. Optical microscopy images (objective lens with magnification of 10×) (left) and SEM micrographs (right) of Ch films 1.0 w/v% containing 4-AAB (a) 2.5 wt%, (b**)** 5.0 wt%, (c) 7.5 wt% and (d) 10 wt%. The bars shown in the images of the left and right panels correspond to 20 µm and 5 µm, respectively.

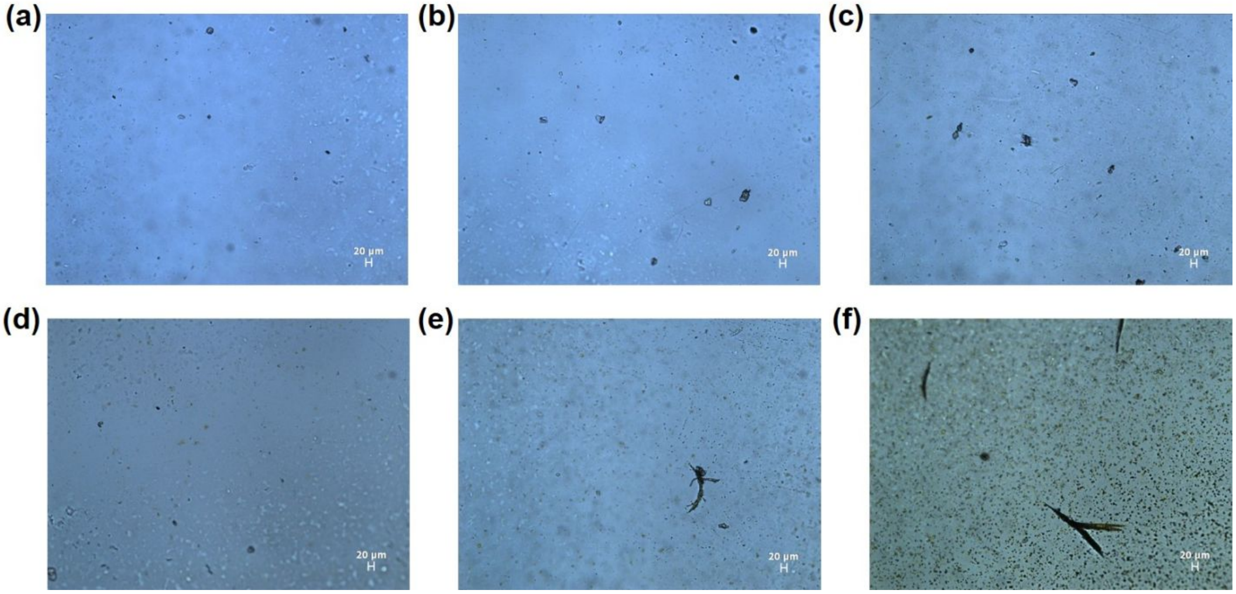

Figure S6. Optical microscopy images (objective lens with magnification of 10×) of Ch films 1.0 w/v% containing 4-AAB (a) 0.05 wt%, (b) 0.1 wt%, (c) 0.3 wt%, (d) 0.5 wt%, (e) 0.7 wt% and (f) 0.9 wt%. The bars shown in the images correspond to 20 µm. No fluorescence was observed for these samples.

Despite the presence of some particles, Ch films containing PTPh displayed a more homogeneous surface (Figure S7). These particles showed emission in the fluorescence optical microscopy images, suggesting the formation of PTPh aggregates within the Ch matrix. As the PTPh concentration was increased, the quantity and size of these particles increased. Additionally, SEM images show a rougher surface for high concentrations of conjugated polymer (7.5 and 10 wt%). Therefore, PTPh demonstrates partial miscibility and good compatibility with the Ch matrix.

When 4-AAB (≤ 1 wt%) was introduced into the Ch/PTPh (2.5 wt%) film a single-phase surface was observed (Figure S8), suggesting the greater dispersibility of 4-AAB when PTPh is integrated into the Ch matrix. As the 4-AAB concentration was raised a rougher surface was observed and the PTPh emission intensity decreased (Figure S8 – left).

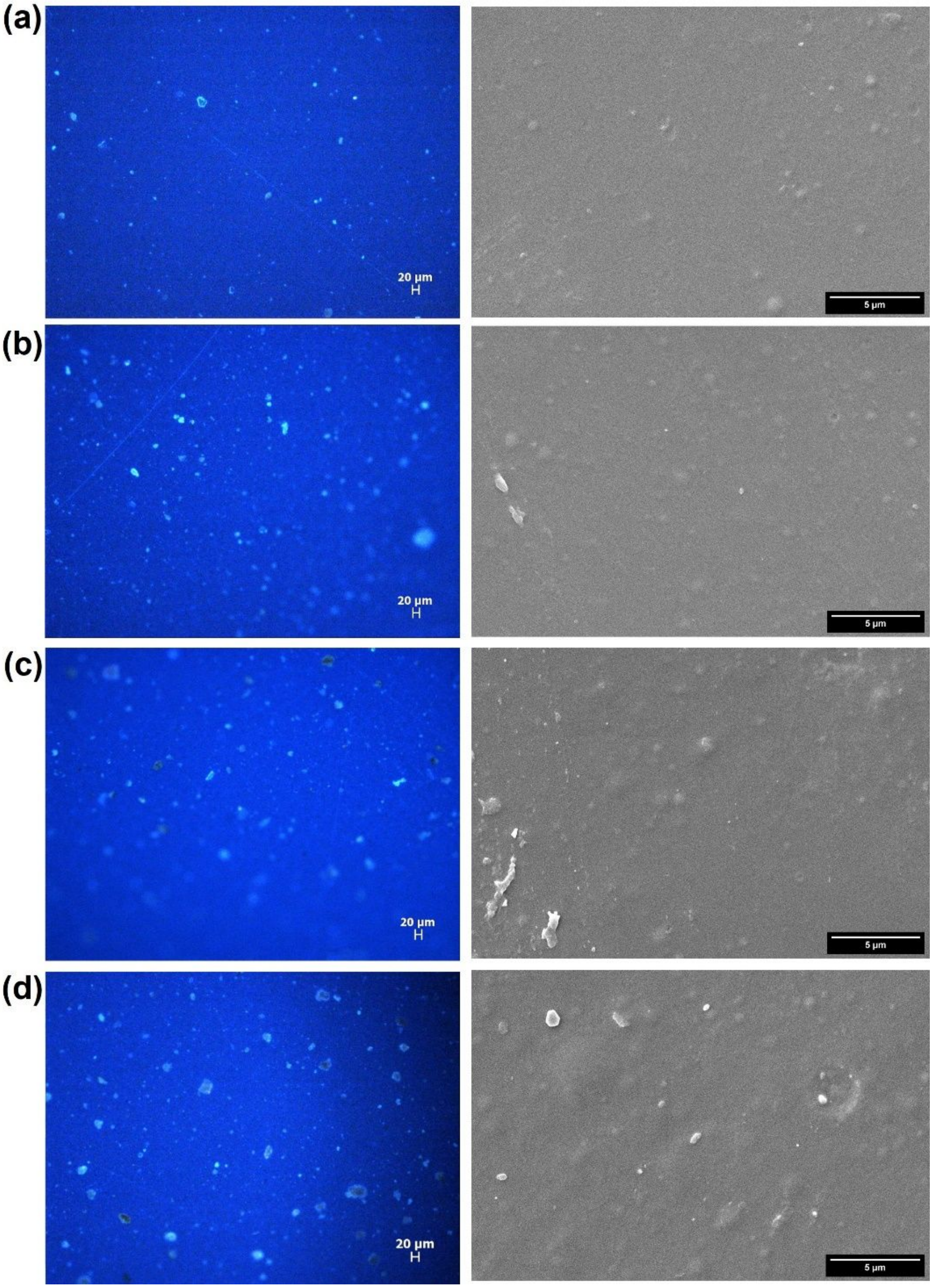

Figure S7. Optical microscopy images (objective lens with magnification of 10×) (left) and SEM micrographs (right) of Ch films 1.0 w/v% containing PTPh (a) 1.0 wt%, (b) 5.0 wt%, (c) 7.5 wt% and (d) 10 wt%. The images from optical microscopy were obtained with a DAPI filter (λ_{ex} = 365 nm) and the same settings were used on the microscope enabling the comparison of intensities for the different films. The bars shown in the images of the left and right panels correspond to 20 µm and 5 µm, respectively.

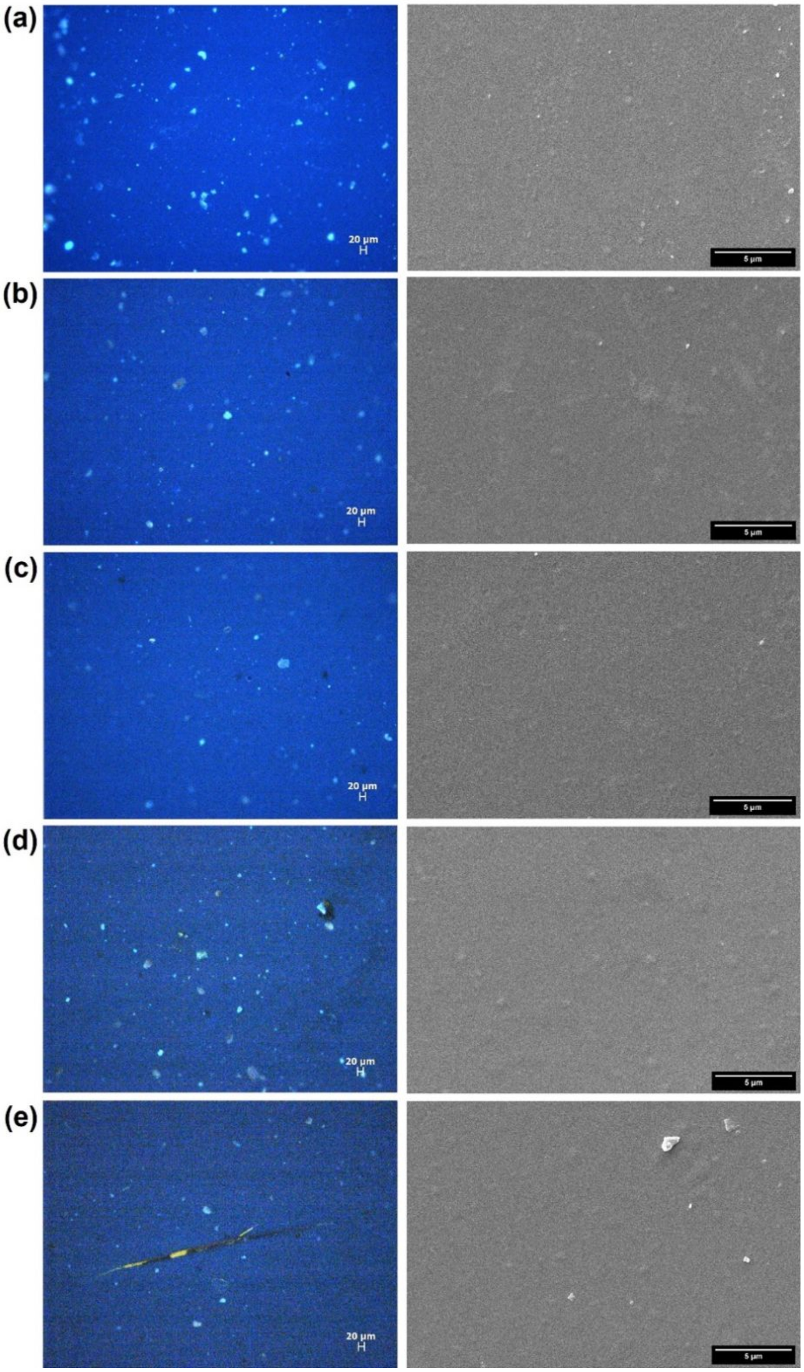

Figure S8. Optical microscopy images (objective lens with magnification of 10×) (left) and SEM micrographs (right) of Ch films 1.0 w/v% containing PTPh (2.5 wt%) and 4-AAB (a) 0.1 wt%, (b) 0.3 wt%, (c) 0.5 wt%, (d) 0.7 wt% and (e) 0.9 wt%. The images from optical microscopy were obtained with a DAPI filter (λ_{ex} = 365 nm) and the same settings were used on the microscope enabling the comparison of intensities for the different films. The bars shown in the images of the left and right panels correspond to 20 µm and 5 µm, respectively.

3. Photophysics of Ch/PTPh, Ch/4-AAB and Ch/PTPh/4-AAB films

Pristine Ch films absorb in the UV region up to 400 nm (Figure S9). The absorption spectrum of the pristine Ch film was subtracted from the spectra of all films containing PTPh and/or 4-AAB to obtain the spectra of the conjugated polymer and/or of 4-AAB.

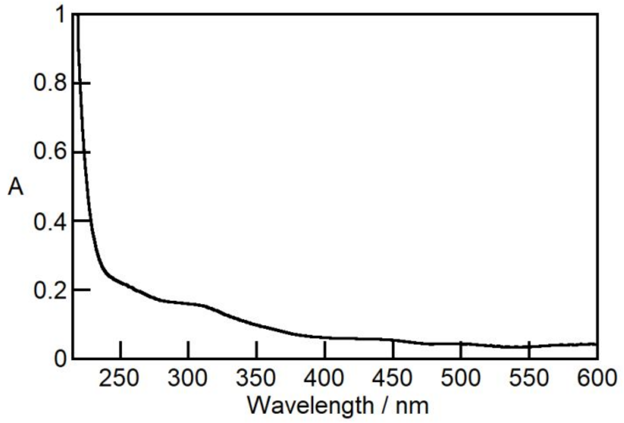

Figure S9. Absorption spectrum of a Ch film 1.0 wt%.

PTPh solubilized in acetonitrile absorbs at 304 nm and emits at 384 nm (Figure S10).

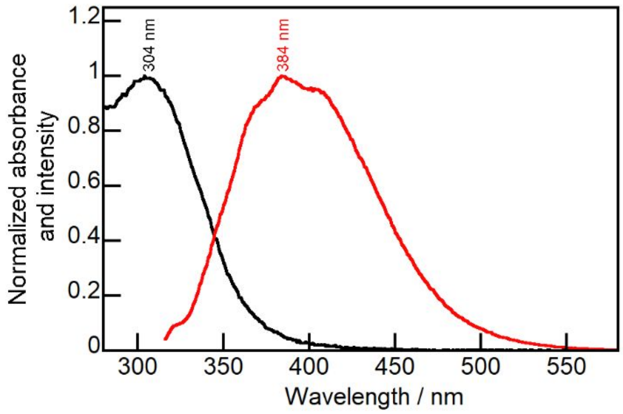

Figure S10. Normalized absorption (black) and emission (red, λ_{ex} = 304 nm) spectra of PTPh (0.021 mg mL^{-1}) in acetonitrile solution.

Exciting a pristine Ch film at 400 nm revealed a broad emission around 460 nm, related to the emission from impurities in Ch (Figure S11). At this excitation wavelength 4-AAB has its absorption maximum. The addition of 4-AAB led to a decrease of the emission intensity of the Ch impurities without the appearance of any new emission bands (Figure S11). This result is consistent with an inner-filter effect where 4-AAB absorbs (Figure 3 in the paper) the emitted light from the Ch impurities. The lack of any new emission band confirmed the non-fluorescent nature of 4-AAB in the films.

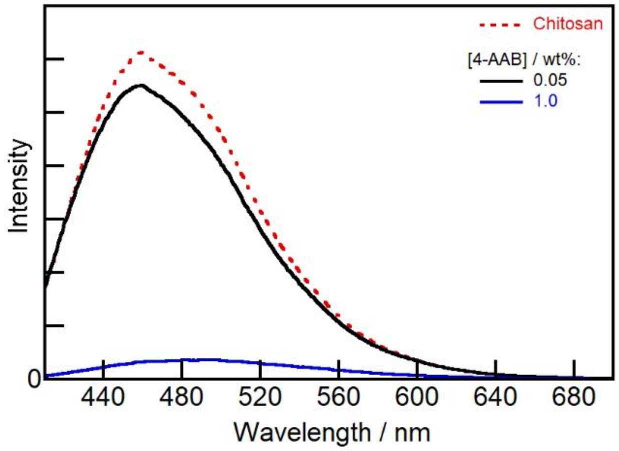

Figure S11. Emission spectra (λ_{ex} = 400 nm) of a pristine Ch film (1.0 w/v%) (red dotted line) and of a film containing 0.05 or 1.0 wt% of 4-AAB.

In the presence of PTPh in the Ch/PTPh films, the emission spectrum with an excitation at 400 nm corresponds to a combination of the emission from PTPh and the emission of impurities in Ch as the emission intensity is higher than for the pristine Ch film (Figure S12). Increasing the 4-AAB concentration in the Ch/PTPh film resulted in a more pronounced decrease in intensity compared to the decreased observed in the absence of PTPh and resulted in a shift in the emission spectrum to longer wavelengths (Figure S12– inset). This result is consistent with an inner filter effect by 4-AAB on the emission of both PTPh and the impurities from Ch because 4-AAB absorbs strongly between 400 and 500 nm.

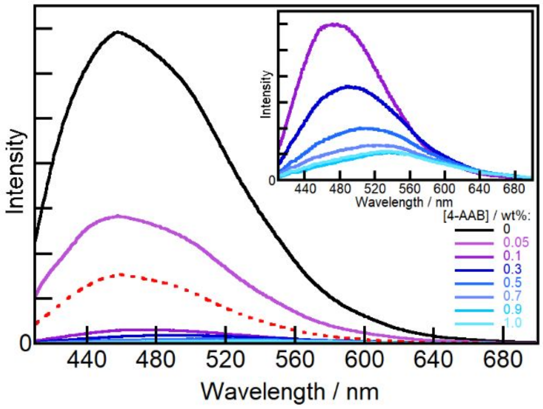

Figure S12. Emission spectra (λ_{ex} = 400 nm) for Ch films 1.0 w/v% containing PTPh (2.5 wt%) with increasing concentrations of 4-AAB (0–1.0 wt%). Inset: expanded intensity scale for spectra containing 0.1 wt% and higher 4-AAB concentrations. The emission from a pristine Ch film 1.0 w/v% film is shown for comparison (red dotted line).

The color of the emission from the Ch/PTPh film was obtained from the analysis of the emission spectrum and is represented in the CIE 1931 chromaticity diagram (Figure S13). The color for the emission of the film was in the blue region and remained the same for all PTPh concentrations, indicating the good dispersion and stability of PTPh within the Ch matrix upon increasing the concentration of the conjugated polymer. The values of the chromaticity coordinates for the emission of the Ch/PTPh and Ch/PTPh/4-AAB films were obtained from the emission spectra of the films using the ColorCalculator v7.77 (Osram Sylvania) software (Table S3).

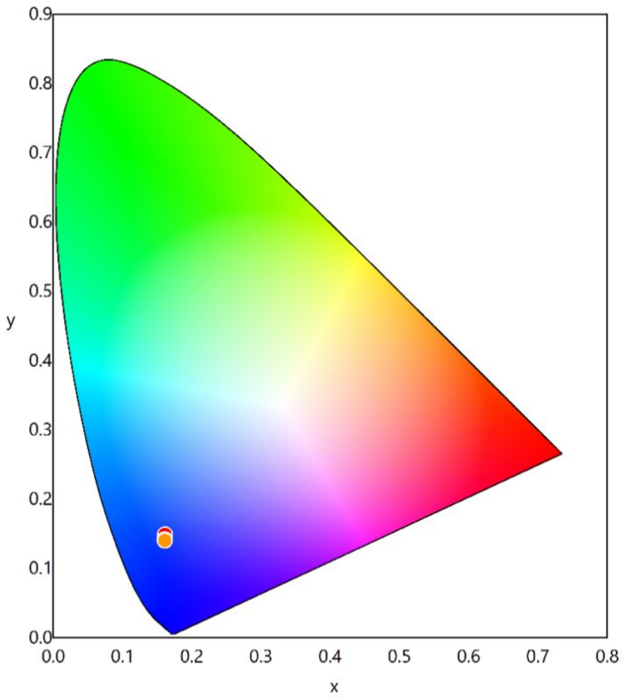

Figure S13. CIE 1931 chromaticity diagram for the Ch films 1.0 w/v% containing PTPh 1.0 wt% (black circle), 2.5 wt% (red circle), 5.0 wt% (green circle), 7.5 wt% (blue circle) and 10 wt% (orange circle). The circles that are not visible are covered by the visible ones.

Table S3. Values of the chromaticity coordinates for the 1.0 w/v% Ch films containing PTPh (1.0–10 wt%) and PTPh (2.5%)/4-AAB (0.05–1.0 wt%).

Sample	x	y
Ch/PTPh (1.0 wt%)	0.1613	0.1461
Ch/PTPh (2.5 wt%)	0.1616	0.1487
Ch/PTPh (5.0 wt%)	0.1609	0.1417
Ch/PTPh (7.5 wt%)	0.1613	0.1418
Ch/PTPh (10 wt%)	0.1613	0.1397
Ch/PTPh (2.5 wt%)/4-AAB (0.05 wt %)	0.1650	0.1536
Ch/PTPh (2.5 wt%)/4-AAB (0.1 wt %)	0.1729	0.1672
Ch/PTPh (2.5 wt%)/4-AAB (0.3 wt %)	0.1898	0.2182
Ch/PTPh (2.5 wt%)/4-AAB (0.5 wt %)	0.2111	0.2731
Ch/PTPh (2.5 wt%)/4-AAB (0.7 wt %)	0.2234	0.2994
Ch/PTPh (2.5 wt%)/4-AAB (0.9 wt %)	0.2446	0.3458
Ch/PTPh (2.5 wt%)/4-AAB (1.0 wt %)	0.2347	0.3235

4. Photoisomerization of 4-AAB in Ch and Ch/PTPh films

The small molecule 4-AAB undergoes photoisomerization from the *trans*- to the *cis*-isomer when irradiated in an acetonitrile solution (Figure S14). Irradiation led to a decrease within 3 min of the absorbance at 382 nm and the appearance of new absorption bands at 345 and 436 nm. The *cis*-to-*trans* isomerization occurs thermally in absence of any irradiation (Figure S14 inset).

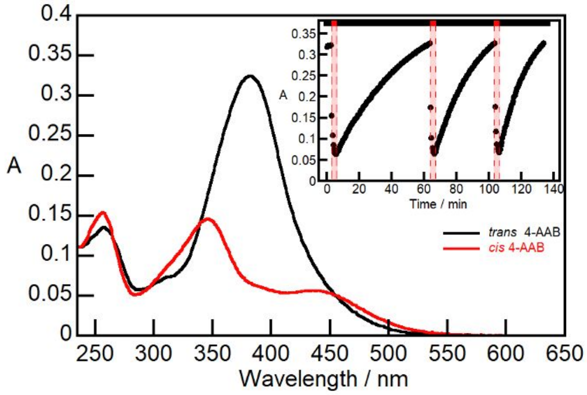

Figure S14. Photoisomerization of 4-AAB (0.17 μM) in an acetonitrile solution. Absorption spectra before (*trans* isomer, black) and after (*cis* isomer, red) UV irradiation (λ_{ex} = 365 nm). Inset: Changes in absorbance at 382 nm over time in the presence (red highlight) and absence of UV irradiation at 365 nm over 3 cycles.

The *trans*-to-*cis* photoisomerization also occurs in Ch/4-AAB and Ch/PTPh/4-AAB films leading to a decrease in absorbance at 400 nm, which corresponds to the 4-AAB absorption maximum (Figure S15). Very small changes in the PTPh absorbance (312 nm) were observed (Figure S15b), confirming that the changes of the film's absorption spectra are due only to changes in the 4-AAB absorption. If PTPh decomposed during the irradiation, a decrease of the absorbance at 312 nm would have been observed. When 4-AAB was incorporated into a Ch film, a slower *cis*-to-*trans* isomerization was observed compared to this reaction in solution (Table S4).

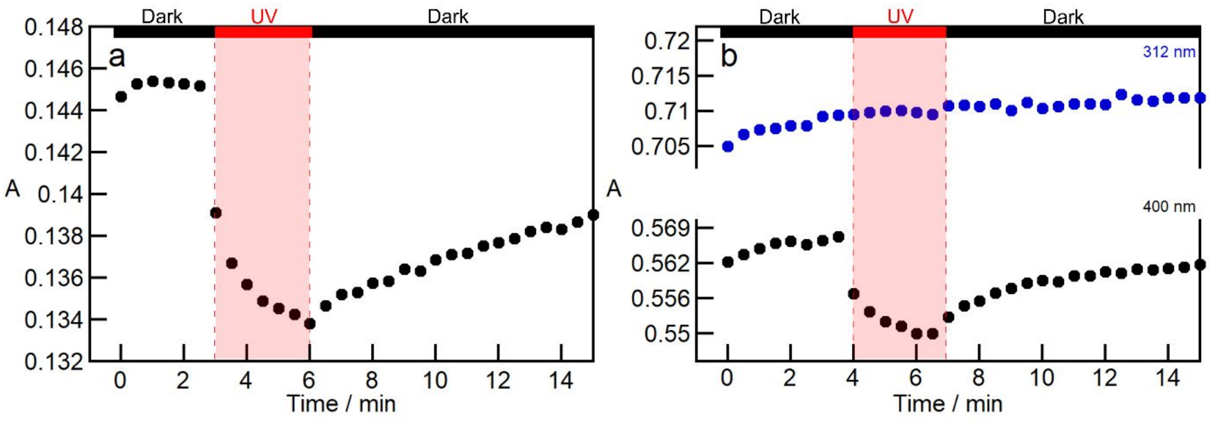

Figure S15. Changes in absorbance values (400 nm (black), 312 nm (blue)) during the first 15 min in the presence (red highlight) and absence of UV light at 365 nm for Ch 1.0 w/v% films containing (a) 4-AAB (0.3 wt%) and (b) PTPh (2.5 wt%)/4-AAB (0.3 wt%).

Table S4. Estimated time of completion of the *cis*-to-*trans* isomerization of 4-AAB (0.17 µM) in an acetonitrile solution, and for the Ch films 1.0 w/v% containing 4-AAB (0.3 wt%) in absence and presence of PTPh (2.5 wt%).[a]

Sample	Completion time of cis-to-trans isomerization / min		
	1st cycle	2nd cycle	3rd cycle
4-AAB in acetonitrile (0.17 µM)	60	40	30
Ch/4-AAB (0.3 wt%)	100	100	100
Ch/PTPh (2.5 wt%)/4-AAB (0.3 wt%)	30	40	40

[a], this experiment was performed once and the estimated precision of the estimated completion times is ± 5 min. The estimated completion time corresponds to either the time the original absorbance before irradiation was reached or to the time the absorbance reached a plateau.

5. 4-AAB adsorption into Ch/PTPh films

Preliminary adsorption experiments of 4-AAB in aqueous solution into Ch/PTPh (2.5 wt%) films were carried out. The Ch/PTPh films were immersed into a 50 µM 4-AAB aqueous solution for 24 h. After adsorption of 4-AAB, the Ch/PTPh film loses its bright blue emission, showing that the film can be used to detect azocompounds present in an aqueous solution.

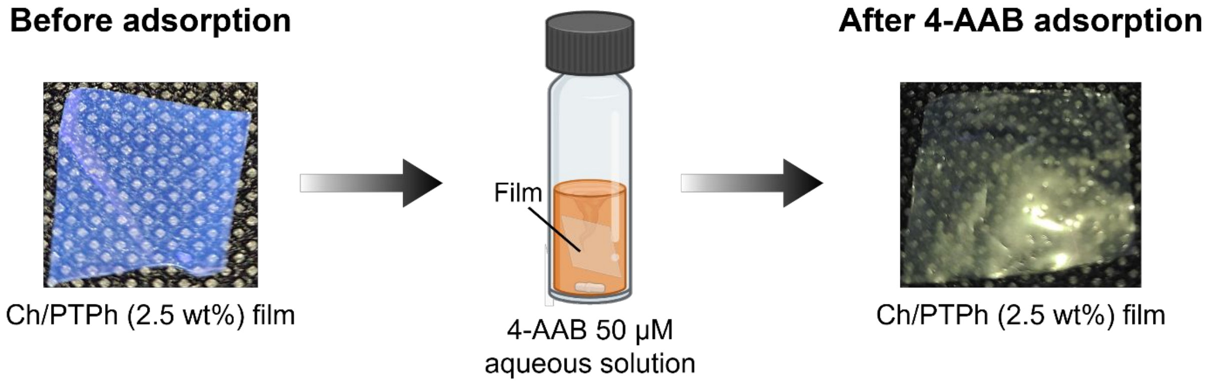

Scheme S1. Schematics for the adsorption experiment of 4-AAB into a Ch/PTPh (2.5 wt%) film. The photos show the emission (λ_{ex} = 312 nm) from dry films before (left) and 24 h after (right) immersion into the aqueous 4-AAB solution.

Adsorption experiments of 4-AAB into pristine Ch films were performed following the same methodology. The concentration of 4-AAB in the aqueous solution was monitored by collecting the absorption spectra of this solution (Figure S16). The decrease of the 4-AAB absorbance suggests that 4-AAB was adsorbed into the Ch film. Similar results were observed for 4-AAB adsorption into Ch/PTPh films, indicating that the presence of the conjugated polymer does not impact the adsorption capacity of Ch films (Table S5).

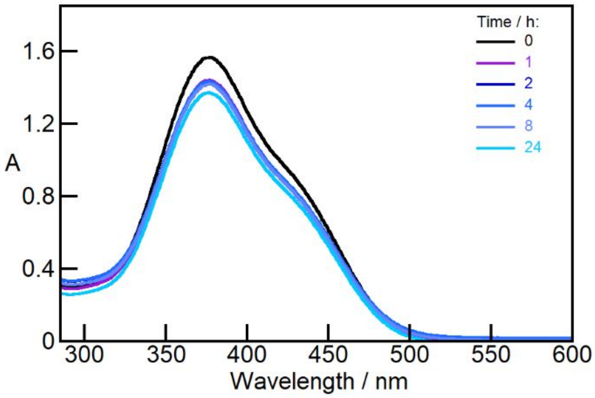

Figure S16. Absorption spectra of a 4-AAB (50 μM) solution in water exposed for different times to a pristine Ch 1.0 w/v% film.

Table S5. Absorbance or differences in absorbance values at 376 nm of the 4-AAB aqueous solutions before and after the 24 h immersion of Ch (1.0 wt%)/PTPh (2.5 %wt) or Ch (1.0 wt%) films.

**Film**	**Time**	**A_{376}**
Ch/PTPh	Before immersion	1.564
	After immersion	1.383
	ΔA	0.181
Ch	Before immersion	1.564
	After immersion	1.374
	ΔA	0.190

REFERENCES

(1) Akira Suzuki. Recent Advances in the Cross-Coupling Reactions of Organoboron Derivatives with Organic Electrophiles. *J. Organomet. Chem.* **1999**, *576*, 147–168.

(2) Menandro, A. S.; Parolin, G. A.; Barbosa, C. G.; Faez, R.; Péres, L. O. New Strategy to Prepare Luminescent Blend by Spin Coating. *Macromol. Symp.* **2019**, *383*, 1–5.
